# High efficacy of particle beam therapies against tumors under hypoxia and prediction of the early stage treatment effect using 3'-deoxy-3'-[^18^F]fluorothymidine positron emission tomography

**DOI:** 10.1007/s12149-023-01877-2

**Published:** 2023-10-19

**Authors:** Akira Makino, Kyo Kume, Tetsuya Mori, Tetsuya Tsujikawa, Tatsuya Asai, Hidehiko Okazawa, Yasushi Kiyono

**Affiliations:** 1 Biomedical Imaging Research Center, University of Fukui, 23-3 Matsuoka-Shimoaizuki, Eiheiji-Cho, Yoshida-Gun, Fukui, 910-1193 Japan; 2 Life Science Innovation Center, University of Fukui, 9-1 Bunkyo-3, Fukui-Shi, Fukui, 910-8507 Japan; 3 The Wakasa Wan Energy Research Center, 64-52-1 Nagatani, Tsuruga-Shi, Fukui, 914-0192 Japan; 4 Graduate School of Engineering, University of Fukui, 9-1 Bunkyo-3, Fukui-Shi, Fukui, 910-8507 Japan

**Keywords:** Positron emission tomography, 3'-Deoxy-3'-[^18^F]fluorothymidine (^18^F-FLT), Hypoxia, Particle beam therapy

## Abstract

**Objective:**

Compared with radiation therapy using photon beams, particle therapies, especially those using carbons, show a high relative biological effectiveness and low oxygen enhancement ratio. Using cells cultured under normoxic conditions, our group reported a greater suppressive effect on cell growth by carbon beams than X-rays, and the subsequent therapeutic effect can be predicted by the cell uptake amount of 3'-deoxy-3'-[^18^F]fluorothymidine (^18^F-FLT) the day after treatment. On the other hand, a hypoxic environment forms locally around solid tumors, influencing the therapeutic effect of radiotherapy. In this study, the influence of tumor hypoxia on particle therapies and the ability to predict the therapeutic effect using ^18^F-FLT were evaluated.

**Methods:**

Using a murine colon carcinoma cell line (colon 26) cultured under hypoxic conditions (1.0% O_2_ and 5.0% CO_2_), the suppressive effect on cell growth by X-ray, proton, and carbon irradiation was evaluated. In addition, the correlation between decreased ^18^F-FLT uptake after irradiation and subsequent suppression of cell proliferation was investigated.

**Results:**

Tumor cell growth was suppressed most efficiently by carbon-beam irradiation. ^18^F-FLT uptake temporarily increased the day after irradiation, especially in the low-dose irradiation groups, but then decreased from 50 h after irradiation, which is well correlated with the subsequent suppression on tumor cell growth.

**Conclusions:**

Carbon beam treatment shows a strong therapeutic effect against cells under hypoxia. Unlike normoxic tumors, it is desirable to perform ^18^F-FLT positron emission tomography 2–3 days after irradiation for early prediction of the treatment effect.

**Graphical Abstract:**

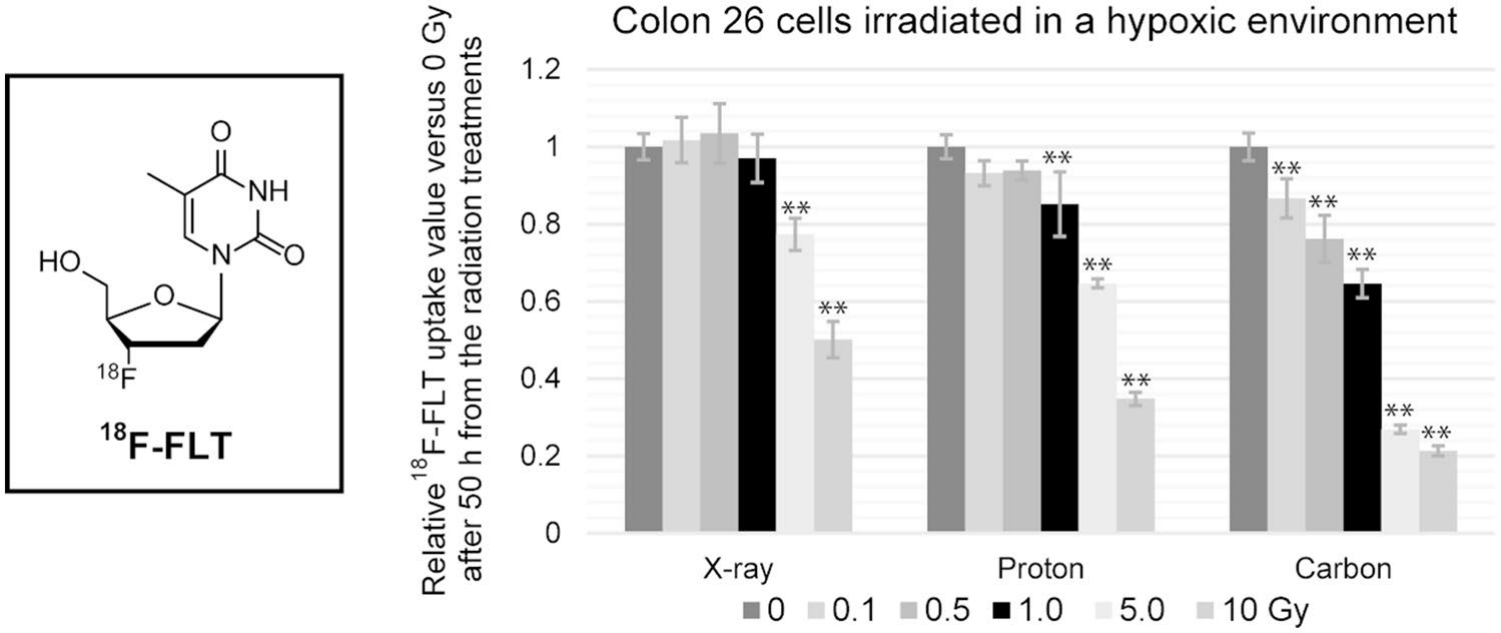

## Introduction

External radiation therapy using photon beams is a major tumor treatment widely used in the clinic. Irradiation of the targeted tumor region damages biomolecules, such as nucleic acids, leading to cell death. The cell damage effect is categorized as direct or indirect, the latter of which is due to ionization and/or excitation of intracellular water and oxygen molecules produced by radiation. Recently, positively charged particle beams, such as proton and carbon beams, have also been utilized for therapy. [1, 2, 3, 4] This is because particle beams have the advantage of exhibiting a Bragg peak, selectively releasing their energy to the targeted tumor region deep inside the body. [5] Additionally, the energy radiation loss per unit length, which is known as linear energy transfer, is high. Therefore, compared with radiation therapy using photon beams, therapies using particles, especially carbon ions have a high relative biological effectiveness (RBE) and low oxygen enhancement ratio (OER). [6].

Our group evaluated the therapeutic effect of particle therapies using a murine colon carcinoma cell line (colon 26). [7] Cultured cells and transplanted tumor model mice were irradiated with X-ray, proton, and carbon beams, and the therapeutic effects were evaluated by counting cell numbers and measuring tumor size. Further, on the day after irradiation, the effect of irradiation on cell function was evaluated using 2-deoxy-2-[^18^F]fluoro-d-glucose (^18^F-FDG) and 3'-deoxy-3'-[^18^F]fluorothymidine (^18^F-FLT), which are positron emission tomography (PET) tracer developed as a glucose metabolism and a cell proliferation marker, respectively [8, 9, 10, 11]. Importantly, glucose metabolism does not show significant difference, but cell proliferation is decreased the day after irradiation. This might be caused by inflammatory response at the radiation sites, which makes it difficult to detect the therapeutic effect by ^18^F-FDG. On the other hand, decreased cell uptake and tumor accumulation of ^18^F-FLT in in vitro and in vivo studies were strongly correlated with subsequent suppression of cell and tumor growth, respectively. Unlike the current approach for predicting treatment effects by observing tumor morphological changes, ^18^F-FLT PET performed soon after irradiation can be used to predict extremely early stage therapeutic effects.

At the tumor site, high levels of energy and oxygen are consumed due to active growth of tumor cells. To supply energy and oxygen, which are essential for tumor cell proliferation, angiogenesis is induced, but sometimes it cannot keep up with tumor growth, thus inducing a local hypoxic environment. [12, 13, 14] As discussed above, radiation beams with low RBE have high OERs; that is, an indirect effect of radiation is considered important for tumor cell growth suppression. [15] On the other hand, particle beam therapies with a low OER show advantages as tumor treatments in a hypoxic environment. Our previous in vitro evaluation was performed under normoxic conditions, and therefore, it is not clear whether the decreased ^18^F-FLT uptake in tumor cells cultured under hypoxic conditions is also correlated with subsequent cell growth suppression. Using tumor cells cultured under hypoxic conditions, this study aims to show the superiority of particle beam therapy and the role of tumor cell functional changes detected by ^18^F-FLT PET as a predictor of irradiation efficacy.

## Materials and methods

### Cell culture

The murine colon carcinoma cell line colon 26, obtained from the Cell Resource Center for Biomedical Research, Tohoku University (Miyagi, Japan), was used in the experiments. In a 75 cm^2^ vent-cap treated cell culture flask (Corning, NY, USA), the cells were cultured in Dulbecco’s modified Eagle’s medium, Nutrient Mixture F-12 (Thermo Fisher Scientific, MA, USA), supplemented with 10% fetal bovine serum (Thermo Fisher Scientific) and 1% penicillin/streptomycin (Thermo Fisher Scientific) at 37°C under 20% O_2_ and 5.0% CO_2_.

Cells were subcultured in a 12.5 cm^2^ plug seal-cap treated cell culture flask (Corning) 3 days prior to irradiation and were incubated at 37°C under normoxic (20% O_2_ and 5.0% CO_2_) conditions for 2 days. Since particle beams were emitted from the horizontal direction, the medium was added up to the neck of the flask, and then, the cells were cultured in a hypoxic (1.0% O_2_ and 5.0% CO_2_) environment with the flask in the upright position for 1 day. After irradiation, the cells were returned to normoxic conditions. While the flasks were in the incubator, the caps were loosened to ventilate the flask interior.

### Western blotting

After adding sample buffer (91 µL) to cells washed with PBS(-), the cells were collected using a scraper and then completely disrupted by sonication using the Branson Sonifier SFX250 (Emerson, MI, USA) at 0°C. The cell lysate was heated at 95°C for 5 min and loaded onto a SDS-PAGE gel (FUJIFILM Wako Chemicals, Osaka, Japan) together with a molecular weight marker (Precision PlusProtein™ AllBlue Prestained Protein Standards, Bio-Rad Laboratories Inc., CA, USA). The gel was run at a constant electric current of 0.02 A for 90 min.

Prior to protein transfer, the gel was washed with blotting buffer. The proteins were transferred from the gel to a PVDF membrane (Immobilon-P, Merck Millipore, MA, USA) activated with methanol (1 min) using the Criterion™ blotter (Bio-Rad Laboratories, Inc.) at a constant voltage of 100 V for 1 h. After washing the membrane with TBS-T, it was blocked in 5% skim milk for 1 h, and cleaved into two around 75 kDa.

The proteins above and below 75 kDa on the membrane were incubated with anti-HIF-1α (Novus Biologicals, CO, USA) or rabbit anti-actin beta (Bio-Rad Laboratories, Inc.) antibody overnight. Then, the membrane was washed with TBS-T and incubated with mouse anti-rabbit IgG-HRP (Santa Cruz Biotechnology Inc., TX, USA) as the secondary antibody. Images were taken using the LAS-3000 imager (FujiFilm, Tokyo, Japan) and the Chemi-Lumi One Super luminol-based chemiluminescence assay kit for western blotting (Nacalai Tesque, Kyoto, Japan).

### Irradiation

Cells at 50–60% confluency were irradiated with X-ray, proton, and carbon beams. Upon removing the flasks from the incubator, the caps were tightly sealed, and the flasks were stored in a styrofoam case at 37°C until the end of the irradiation treatment. X-ray irradiation was performed using the HW-200R (Hitex, Osaka, Japan) with the dose rate fixed at 0.5 Gy/min. Charged particle irradiation was performed using the Wakasa-wan Energy Research Center Multipurpose Accelerator System with Synchrotron and Tandem. [16] The irradiation dose was 0–10 Gy for each radiation beam, and the proton and carbon dose rates were 6 and 20 Gy/min, respectively. After irradiation, the cells were cultured under normoxic conditions.

### ^*18*^*F-FLT synthesis*

^18^F-FLT was synthesized from 3-*N*-*tert*-butoxycarbonyl-5'-*O*-dimethodxytrityl-3'-*O*-nosylthymidine (ABX, Radeberg, Germany) using the TRACERlab MX-FDG synthesizer (GE Healthcare, IL, USA). ^18^F-fluoride was produced via the ^18^O(p,n)^18^F reaction from > 98% enriched ^18^O-H_2_O (Taiyo Nippon Sanso, Tokyo, Japan) using the radioisotope delivery system eclipse^RD/HP^ medical cyclotron (Siemens, Munich, Germany). The ^18^F-FLT radiochemical yield and purity were 11.2 ± 4.1% and 98.6 ± 0.6%, respectively (*n* = 6).

### Cell counting

At 1–4 days after irradiation, the medium was removed, and the cells were washed with PBS (2.0 mL) three times. Cells were detached by incubating in trypsin–EDTA (0.20 mL) (Thermo Fisher Scientific) for 5 min, and then, the reaction was stopped by applying medium (0.20 mL). The cell suspension was pipetted thoroughly, and the viable cells were counted by 0.4% trypan blue (Thermo Fisher Scientific) staining using the Countess-II FL automatic cell counter (Thermo Fisher Scientific).

### ^*18*^*F-FLT cell uptake*

On the day after irradiation, the medium was changed to fresh medium containing ^18^F-FLT (2.0 mL), with the radioactivity set to 1.0 MBq/flask. The flasks were incubated at 37°C under 20% O_2_ and 5.0% CO_2_ for 1 h, and then, the medium was removed. The cells were washed with ice-cold PBS (2.0 mL) three times, after which 0.1 mol/L sodium hydroxide solution (2.0 mL, Nacalai Tesque) was added, and the cells were lysed by pipetting thoroughly. The radioactivities of the cell lysate (0.20 mL, *n* = 3) and ^18^F-FLT-containing medium used for the cell uptake study as controls were measured using the Wallac 1480 Wizard 3 gamma counter (Perkin Elmer, MA, USA). In parallel, the protein concentration in the cell lysate was determined using the Pierce™ BCA Protein Assay Kit (Thermo Fisher Scientific) and then measuring the absorbance at 562 nm using the SpectraMax M5 plate reader (Molecular Devices, CA, USA); a calibration curve was prepared from albumin standards. The percentage of ^18^F-FLT cell uptake was normalized to the protein amount (mg).

### Statistical analysis

Statistical analyses were performed using GraphPad Prism 7 (GraphPad Software, MA, USA). Differences between the untreated control and irradiation groups were analyzed by one-way ANOVA with adjustment using the Bonferroni method. A *P* value less than 0.05 was considered statistically significant.

## Results

### Cells cultured under hypoxic conditions

**Fig. 1:**
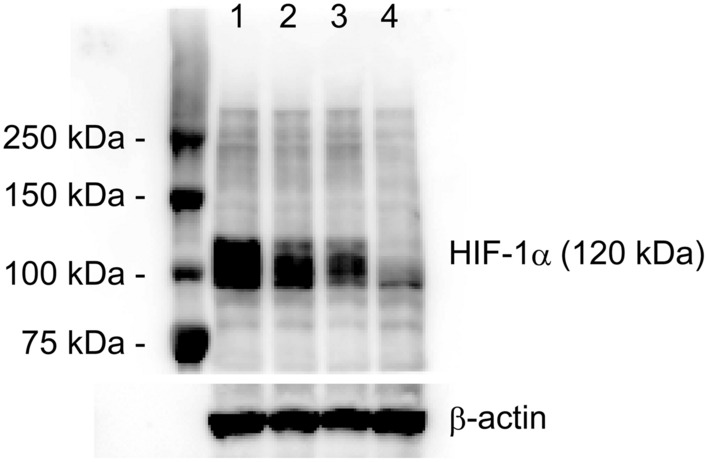
Representative Western blot images for HIF-1α. β-Actin was used as the internal control. After blotting to a PVDF membrane, the blot was divided in two under 75 kDa and stained with anti-HIF-1α and anti-β-actin antibodies, respectively. Cells cultured under hypoxic conditions are shown in Lanes 1–3, and those under normoxic conditions in Lane 4. Lanes 1 and 2 represent cells cultured under the same conditions as those in the irradiation experiments (upright state). Lane 1 represents cells when the cell flask was taken out from the hypoxic chamber. Lane 2 represents cells when the flask was additionally stored in a heat-retaining case for 1 h thereafter in the same manner with the radiation experiment. Lanes 3 and 4 represent cells cultured in flask under normal horizontal position with the adherent cell side down

To confirm that a hypoxic environment was maintained during the experiment, the expression level of the hypoxia-inducible factor (HIF-1), which is a well-known transcription factor activated under the hypoxic state, was evaluated by Western blotting (Fig. 1). Lanes 3 and 4 in Fig. 1 represent control cells cultured in hypoxic and normoxic chambers, respectively. Cells cultured in the hypoxic chamber (Lane 3) showed a clear HIF-1α band at 120 kDa, while those cultured in the normoxic chamber (Lane 4) showed a weak band. In this study, it was necessary to close the cap of the flask taken out of the hypoxic chamber and put them in a heat-retaining case for 1 h until irradiation. Then, the state of cells cultured in the same manner with radiation experiments was examined immediately after being removed from the chamber (Lane 1) and after being stored in the case for 1 h (Lane 2). Both cells showed HIF-1α bands, confirming hypoxic state was maintained.

### Cell counts

**Fig. 2:**
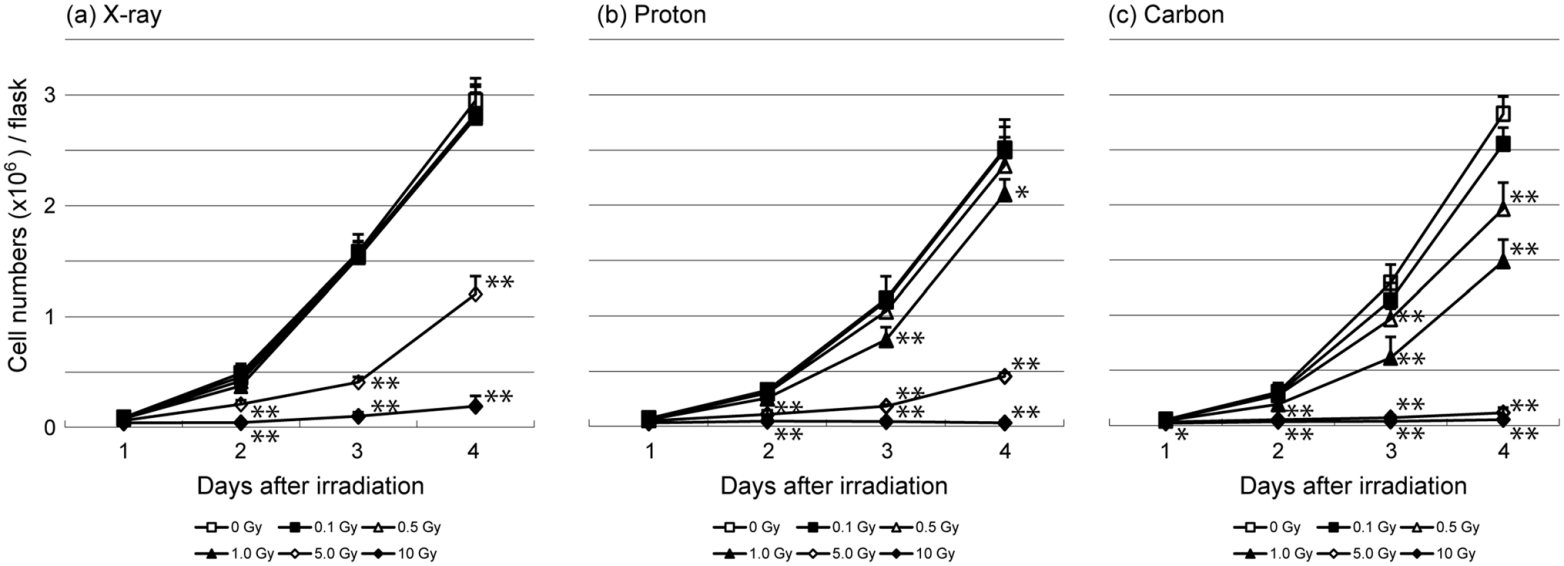
Proliferation curves for colon 26 cells. The cells were treated with (a) X-ray, (b) proton, or (c) carbon beams at doses of 0–10 Gy/flask under hypoxic conditions. Cells were cultured under normoxic conditions after irradiation. *, significant difference compared with the untreated control cells (0 Gy) (* *p* < 0.05, ** *p* < 0.01)

The cells cultured under hypoxic conditions were irradiated with X-ray, proton, or carbon beams at 0.1, 0.5, 1.0, 5.0, and 10 Gy. Then, the irradiated cells were incubated under normoxic conditions for 1–4 days. The cell numbers on days 1–4 after irradiation are shown in Fig. 2. Regardless of the radiation type, cells irradiated with ≥ 5 Gy were significantly decreased in number from day 2 after irradiation compared with the control cells (0 Gy). In addition, cells irradiated with proton (1.0 Gy) and carbon (0.5 and 1.0 Gy) beams were significantly decreased in number from day 3 after irradiation compared with the control cells.

### ^*18*^*F-FLT cell uptake after irradiation*

**Fig. 3:**
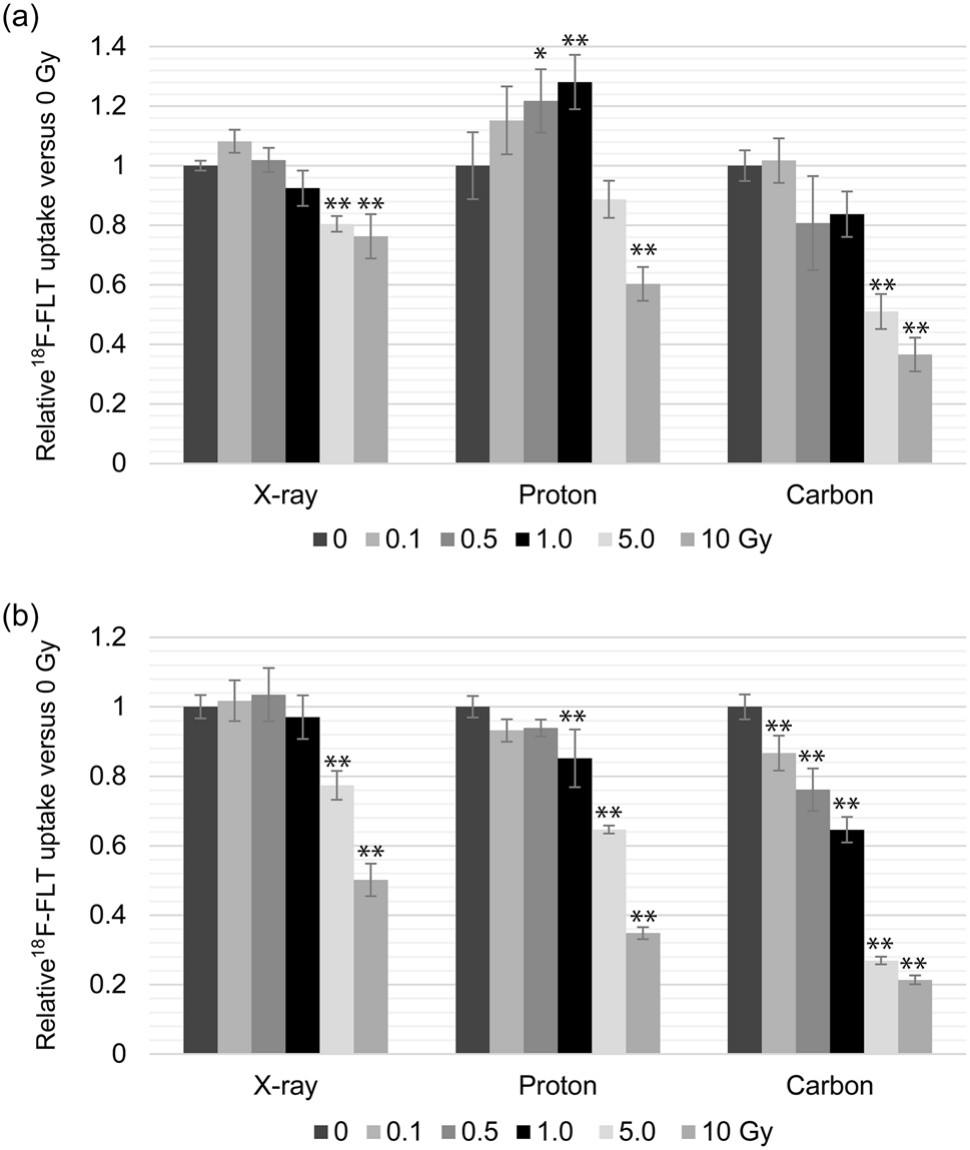
Amount of ^18^F-FLT uptake in colon 26 cells at (a) 26 h and (b) 50 h after irradiation under hypoxic conditions. *n* = 6 for each condition. *, significant difference compared with the untreated control cells (0 Gy) (* *p* < 0.05, ** *p* < 0.01)

Cells were incubated in medium containing ^18^F-FLT at 26 h and 50 h after irradiation, and ^18^F-FLT cell uptake was measured (Table 1). Figure 3 shows the results summarizing the changes in ^18^F-FLT uptake according to the irradiation dose relative to the uptake in the unirradiated control cells (0 Gy).

**Table 1:** ^18^F-FLT uptake in irradiated cells (colon 26) at (a) 26 h and (b) 50 h after radiation treatment under hypoxia

Type of radiation (Gy)	X-ray (%ID/mg protein)	Proton (%ID/mg protein)	Carbon (%ID/mg protein)
(a) 26 h after irradiation			
0	16.0 ± 0.26	13.4 ± 1.50	16.8 ± 0.86
0.1	17.3 ± 0.62	15.4 ± 1.52	17.1 ± 1.25
0.5	16.3 ± 0.64	16.3 ± 1.42	13.5 ± 2.65
1	14.7 ± 0.94	17.1 ± 1.22	14.1 ± 1.28
5	12.8 ± 0.42	11.9 ± 0.83	8.56 ± 0.99
10	12.2 ± 1.18	8.06 ± 0.76	6.14 ± 0.96
(a) 50 h after irradiation			
0	15.7 ± 0.53	16.6 ± 0.51	19.7 ± 0.70
0.1	16.0 ± 0.92	15.5 ± 0.54	17.1 ± 0.99
0.5	16.2 ± 1.20	15.6 ± 0.41	15.0 ± 1.20
1	15.2 ± 0.99	14.2 ± 1.38	12.7 ± 0.72
5	12.1 ± 0.65	10.8 ± 0.19	5.31 ± 0.21
10	7.86 ± 0.74	5.79 ± 0.28	4.21 ± 0.25

Cell uptake values were calculated as the ratio of radioactivity in the cells (% injection dose) divided by the amount of protein (mg protein), rather than cell number. *n* = 6 for each condition.

The uptake amount of ^18^F-FLT at 26 h and 50 h after X-ray irradiation at doses ≥ 5 Gy was significantly decreased compared with unirradiated cells. In the case of proton beams, ^18^F-FLT uptake differed depending on the time after irradiation. At 26 h, ^18^F-FLT uptake was significantly increased at 0.5 and 1.0 Gy, but was decreased at 10 Gy. On the other hand, uptake after 50 h was decreased only at doses above 1.0 Gy. ^18^F-FLT uptake in cells at 26 h and 50 h after receiving carbon ion beams above 5.0 Gy and 0.1 Gy was decreased significantly, respectively.

## Discussion

### Cell culture

Cells prepared for the irradiation experiments were maintained under hypoxic conditions. As shown in Fig. 1, the expression of HIF-1α on Western blotting was higher when the cells were cultured in a flask in the upright position (Lanes 1 and 2) compared with the normal horizontal position (Lane 3). The gas–medium interface area becomes smaller when the flask is cultured upright (Lanes 1 and 2). In the upright position, the oxygen supply in the medium is worse, resulting in a low dissolved oxygen concentration. Also, HIF-1α expression in Lane 2 was approximately 30% higher than that in Lane 1, albeit not significantly (data not shown) (*n* = 3). This might be because the medium occupied at least 90% of the flask volume, leaving almost no air space in the flask. After the lid was closed tightly, the cells used up the dissolved oxygen in the medium, resulting in greater hypoxia.

### Cell counting

**Fig. 4:**
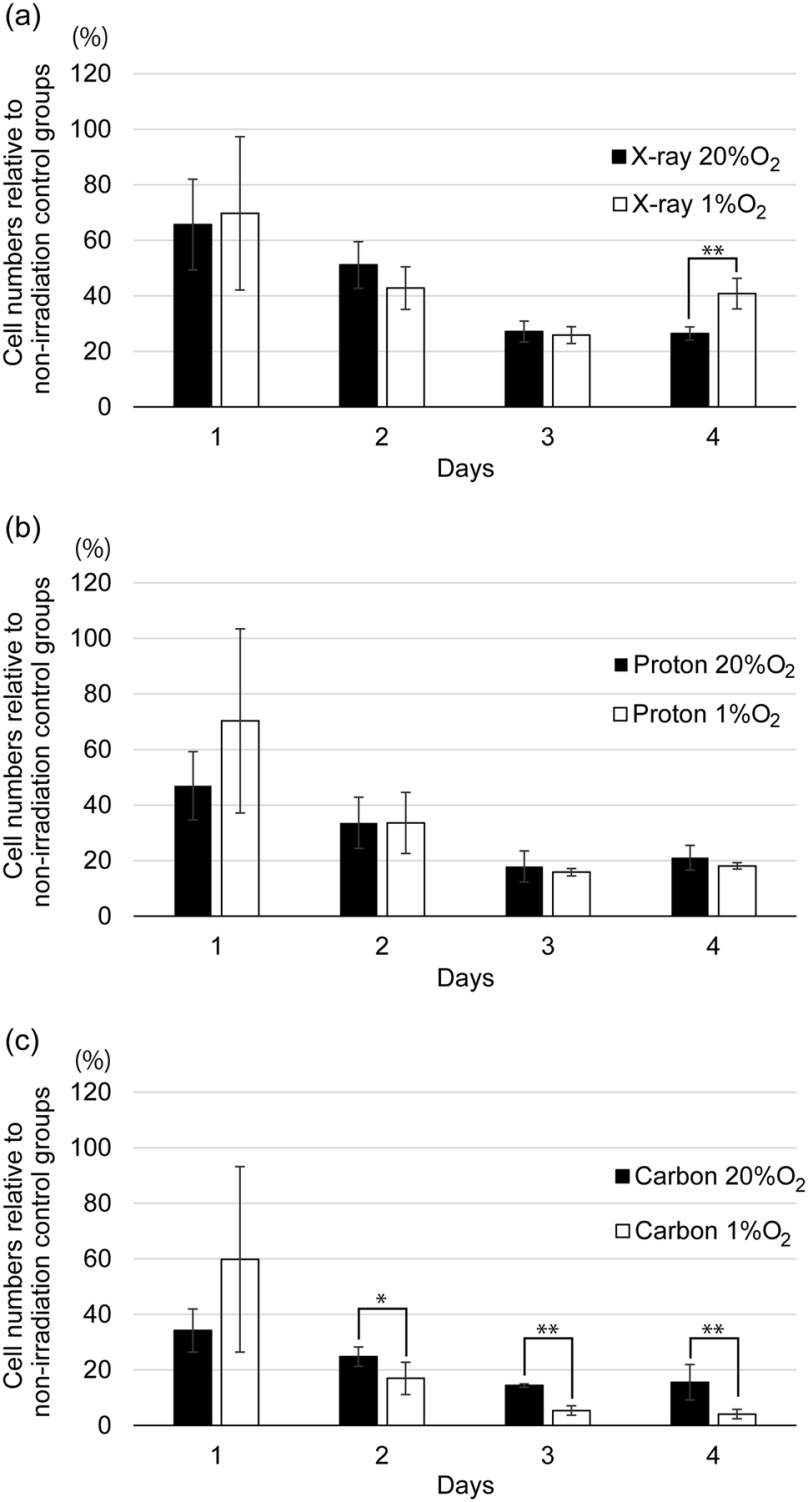
Cell numbers relative to those in the non-irradiation control groups after irradiation with 5 Gy (a) X-ray, (b) proton, or (c) carbon beams under normoxic (20% O_2_) or hypoxic (1% O_2_) conditions (*p < 0.05, **p < 0.01)

Among the three radiation types tested, carbon ions had the greatest suppressive effect on cell growth, which is supported by the higher relative biological effectiveness (RBE) of proton and carbon beams. Charged particle therapies are expected to give better treatment results, even for hypoxic tumors. Figure 4 shows cell growth suppression at different time points in cells cultured under normoxic and hypoxic conditions after irradiation with 5 Gy of each radiation beam. The data for the hypoxic conditions were derived from Fig. 2, and those for the normoxic conditions were from our previous report [7]. In cells irradiated with X-rays (Fig. 4a), there was almost no difference in growth between the hypoxic and normoxic conditions from days 1 to 3, whereas on day 4, cell growth was significantly more suppressed under normoxic than hypoxic conditions. This might be because the cells irradiated with X-rays under hypoxic conditions did not receive an oxygen enhancement effect. On the other hand, with proton beams, the cell culture environment did not influence the irradiation effect (Fig. 4b). Surprisingly, cell growth suppression by carbon beams was stronger under hypoxia. Under hypoxia, the population of cells in the G0/G1 phase increased [17, 18]. It has been reported that chromosomal aberrations and fragments are significantly higher in the G_0_/G_1_ phase than in the G_2_/M phase in cells irradiated with particles with a high linear energy transfer [19]. Together with the ability of particle beams to damage cells directly, carbon ions appear to be superior to X-rays in treating tumors under hypoxic conditions.

### ^*18*^*F-FLT cell uptake after irradiation*

After 26 h from the 5 Gy X-ray, proton- and carbon-beam irradiations, ^18^F-FLT cell uptake cultured in normal oxygen state was decreased about 22%, 23%, and 40% from control (0 Gy), respectively. [7] On the other hand, those cultured in hypoxic state were 20%, 11%, and 49%, respectively, as shown in Table 1. Detailed evaluations in the future will be essential because the transient increase in ^18^F-FLT uptake was observed on cells cultured in hypoxic state, especially in low doses. However, the stronger suppression of ^18^F-FLT cell uptake by carbon beams in cells cultured under hypoxic environment may reflect the oxygen-state-dependent cytocidal effects (Fig. 4c).

^18^F-FLT uptake at 50 h (Fig. 3b) was decreased in an irradiation dose-dependent manner, which coincided with the subsequent suppression of cell growth. When the cells cultured under normoxic conditions were irradiated, ^18^F-FLT uptake was decreased on the day after irradiation [7], but ^18^F-FLT uptake at 26 h (Fig. 3a) showed a temporary increase, especially in cells treated with low-dose radiation (0.1–1 Gy). FLT tumor uptake is thought to be correlated with thymidine kinase-1 (TK1) activity in the thymidine salvage pathway. TK1 is suggested to be a factor that indirectly controls DNA repairs [20], and it has been reported TK1 mRNA level transiently increases by X-ray irradiation [21]. Further, the populations of cells in different cell cycle phases differ between hypoxia and normoxia as discussed above [17, 18]. In this study, we reoxygenated the cells soon after the radiation treatments. The reoxygenated cells re-enter the cell cycle, but the cell cycle phases may continue to be asynchronous for some time after reoxygenation. Because the doubling time of the cells used for this experiment was 22–24 h, biased cell population in S phase is considered to be left after 26 h from the reoxygenation. It has been reported that ^18^F-FLT uptake peaks during the S and G2 phases of the cell cycle [22]. Caused by transient increase of TK1 mRNA level and biased cell cycle due to reoxygenation, the increased tumor uptake after low-dose irradiation (0.1–1.0 Gy), especially with proton beams, may be occurred. To predict the irradiation treatment effect, ^18^F-FLT-PET should be performed 2–3 days after treatment, when the cell cycle has normalized again.

### Limitations of this study

It is not clear yet how radiation treatments affects hypoxic regions of tumors, because tumor local state is thought to change dynamically over time. Therefore, our experimental conditions did not completely mimic radiation therapy for hypoxic tumors. Additionally, this study was performed using only one cell line and a single hypoxic state. The cell hypoxic condition was confirmed by detecting HIF-1 protein, but not by direct measurements of cell culture medium using oxygen meter. Since the degradation speed of HIF-1 is dependent of the cell types, expression levels of HIF-1 might not reflect the real-time hypoxic state.

## Conclusions

Among the three types of radiation tested, carbon beams had the greatest ability to suppress cell growth, especially in cells cultured under hypoxic compared with normoxic conditions. When ^18^F-FLT cell uptake was evaluated at 50 h after irradiation, the reduction in ^18^F-FLT uptake coincided well with the subsequent cell growth suppression. Therefore, ^18^F-FLT is expected to be a predictive marker for radiation therapy effectiveness. Further evaluations are required for clinical application, but ^18^F-FLT examination performed 2–3 days after irradiation may be recommended for predicting the treatment effect.

## Data Availability

The data that support the findings of this study are available from the corresponding authors upon reasonable request.
